# Hemorheological and Glycemic Parameters and HDL Cholesterol for the Prediction of Cardiovascular Events

**DOI:** 10.5935/abc.20150146

**Published:** 2016-01

**Authors:** Sung Woo Cho, Byung Gyu Kim, Byung Ok Kim, Young Sup Byun, Choong Won Goh, Kun Joo Rhee, Hyuck Moon Kwon, Byoung Kwon Lee

**Affiliations:** 1 Division of Cardiology - Department of Internal Medicine - Sanggye Paik Hospital, Inje University College of Medicine, Seoul - Korea; 2 Division of Cardiology - Department of Medicine - Samsung Medical Center, Seoul - Korea; 3 Division of Cardiology - Department of Internal Medicine - Gangnam Severance Hospital - Yonsei University College of Medicine, Seoul - Korea

**Keywords:** Atherosclerosis, Coronary Artery Disease, Blood Sedimentation, Fibrinogen, Cardiovascular Diseases / adverse events

## Abstract

**Background:**

Hemorheological and glycemic parameters and high density lipoprotein (HDL) cholesterol are used as biomarkers of atherosclerosis and thrombosis.

**Objective:**

To investigate the association and clinical relevance of erythrocyte sedimentation rate (ESR), fibrinogen, fasting glucose, glycated hemoglobin (HbA1c), and HDL cholesterol in the prediction of major adverse cardiovascular events (MACE) and coronary heart disease (CHD) in an outpatient population.

**Methods:**

708 stable patients who visited the outpatient department were enrolled and followed for a mean period of 28.5 months. Patients were divided into two groups, patients without MACE and patients with MACE, which included cardiac death, acute myocardial infarction, newly diagnosed CHD, and cerebral vascular accident. We compared hemorheological and glycemic parameters and lipid profiles between the groups.

**Results:**

Patients with MACE had significantly higher ESR, fibrinogen, fasting glucose, and HbA1c, while lower HDL cholesterol compared with patients without MACE. High ESR and fibrinogen and low HDL cholesterol significantly increased the risk of MACE in multivariate regression analysis. In patients with MACE, high fibrinogen and HbA1c levels increased the risk of multivessel CHD. Furthermore, ESR and fibrinogen were significantly positively correlated with HbA1c and negatively correlated with HDL cholesterol, however not correlated with fasting glucose.

**Conclusion:**

Hemorheological abnormalities, poor glycemic control, and low HDL cholesterol are correlated with each other and could serve as simple and useful surrogate markers and predictors for MACE and CHD in outpatients.

## Introduction

Atherosclerosis, thrombosis, and plaque formation are progressive and dynamic consequences of the complex interactions between endothelial dysfunction, inflammation, and hemorheological factors. Hemorheological abnormalities may lead to elevated shear forces at the vascular endothelium by increasing red blood cell (RBC) aggregation and local blood viscosity, promoting endothelial injury.^1^ They may also trigger the rupture of lipid-rich, unstable atherosclerotic lesion, thus leading to thrombus formation and clinical symptoms of acute coronary syndrome.^2,3^ These phenomena are indicated by several hemorheological parameters, including the acute phase reactants erythrocyte sedimentation rate (ESR) and fibrinogen,^1,4^ which are therefore predictors and biomarkers of major adverse cardiovascular events (MACE), acute coronary syndrome, coronary heart disease (CHD), and ischemic stroke.^5-14^

Hemorheological abnormalities such as increased blood and plasma viscosity, enhanced RBC aggregation, and decreased RBC deformability have been described in patients with diabetes mellitus (DM).^15,16^ Chronic complications of diabetes are macro- and micro-vascular dysfunction, which may increase the potential for thrombosis and plaque formation and finally increase cardiovascular mortality. Furthermore, abnormalities in blood rheology are prominent in patients with poor glycemic control.^15^ Indeed, glycated hemoglobin (HbA1c), which is the parameter of glycemic control, has been reported as a predictor and risk factor of MACE, acute coronary syndrome, and CHD in both diabetic and non-diabetic patients.^17-22^ Also, it is well established that low high density lipoprotein (HDL) cholesterol level is a cardiovascular risk factor, negatively correlated with blood viscosity.^23^

Previous studies have reported correlations between hemorheological abnormalities, poor glycemic control, and low HDL cholesterol.^17,24,25^ However, few reports have addressed such relationship by taking into account simultaneously these three risk factors.^25^ In the present study, we investigated the association of hemorheological and glycemic parameters with HDL cholesterol, and the clinical relevance of these factors in the prediction of MACE and CHD in an outpatient population.

## Methods

### Study population and design

From February 2007 to January 2009, 708 stable patients who visited the outpatient department were enrolled in the study and followed until June 2010. Patients with acute or chronic infection, inflammatory disease, liver failure, renal insufficiency, or cancer were excluded from the analysis. Hypertension was considered as the presence of repeated measurements of systolic blood pressure ≥ 140 mmHg and/or diastolic blood pressure ≥ 90 mmHg, or antihypertensive drug treatment. DM was defined as a fasting blood glucose concentration ≥ 126 mg/dL or use of antihyperglycemic drugs. Smoking was assessed by a self-administered questionnaire, with current smoking defined as any smoking within the past year.

Patients were divided into two groups, patients with MACE and patients without MACE. MACE included cardiac death, acute myocardial infarction (AMI), newly diagnosed CHD, and cerebral vascular accident (CVA). Patients with AMI were diagnosed on the basis of clinical presentations, specific electrocardiographic alterations, and serum cardiac enzyme levels. CHD was diagnosed by coronary angiography or coronary computed tomography (CT) angiography. A significantly diseased artery was defined as having ≥ 50% stenosis in at least one of its segments. Patients with CVA were diagnosed on the basis of clinical presentations and cerebral imaging modality such as CT or magnetic resonance imaging.

We received approval from the institutional review board of Inje University Sanggye Paik Hospital, Seoul, Korea to conduct all these analyses. All subjects included gave their informed consent at the time of the examination.

### Blood sampling and preparation

Blood samples were taken by venipuncture after an overnight fast from patients. Serum fasting glucose, total cholesterol, triglyceride, HDL cholesterol, low-density lipoprotein (LDL) cholesterol, blood urea nitrogen (BUN), and creatinine levels were measured using dedicated reagents by automatic chemistry analyzer (AU 5400, Beckman-Coulter, Fullerton, CA, USA). Complete blood count (CBC) was measured using XE-2100 (Sysmex cooperation, Kobe, Japan). ESR was measured by Test-1 analyzer (Alifax, Padova, Italy) and fibrinogen was determined using coagulometric techniques in a CA-1500 autoanalyzer (Sysmex). HbA1c was measured by a high-pressure liquid chromatographic assay (G8 system, Tosoh, Japan). All of the laboratory parameters were determined in the central laboratory of the Inje University Sanggye Paik Hospital.

### Statistical analysis

All data were analyzed using the PASW 18.0 software. Categorical variables were expressed as percentages and continuous variables as means ± standard deviation. Normally distributed continuous variables were compared using unpaired student t-test, and categorical variables were compared using the chi-squared test. Binary multivariate logistic regression analysis was performed, starting with a model including a number of potential confounders (age, gender, hypertension, diabetes, and total cholesterol levels). Spearman rank correlation test was used for correlation analyses of continuous parameters. p value of < 0.05 was considered statistically significant.

## Results

### MACE and baseline clinical characteristics in study population

The mean follow-up period of the study population was 28.5 months, and MACE occurred in 60 patients during this period. Three patients died (one because of sudden cardiac death and two because of heart failure), one patient was admitted due to AMI, 53 patients were newly diagnosed CHD, and 3 patients were admitted due to CVA (Table 1).

**Table 1:** Frequency (%) of events in 60 patients with adverse cardiac events

**Events**	**N (%)**
Death	3 (5%)
Myocardial infarction	1 (1.7%)
Coronary heart disease	53 (88.3%)
Cerebrovascular accident	3 (5%)

The patients with MACE were significantly older and had significantly higher incidence of hypertension and DM compared to patients without MACE. In addition, the patients with MACE had significantly lower levels of hemoglobin, hematocrit, platelet, and HDL cholesterol, while higher levels of ESR, fibrinogen, fasting glucose, and HbA1c compared to patients without MACE. The baseline clinical characteristics of the study population are summarized in Table 2.

**Table 2:** Baseline clinical characteristics of the study population

**Variable**	**MACE (-) (n = 648)**	**MACE (+) (n = 60)**	**p value**
Age (years)	60.2 ± 13.5	66.1 ± 9.7	0.001
Gender (male), n (%)	336 (51.9%)	31 (51.7%)	1.00
Hypertension, n (%)	452 (69.8%)	50 (83.3%)	0.026
Diabetes mellitus, n (%)	125 (19.3%)	21 (35.0%)	0.007
Previous MI, n (%)	76 (11.7%)	8 (13.3%)	0.68
Previous CHD, n (%)	147 (22.7%)	20 (33.3%)	0.08
Smoking, n (%)	242 (37.3%)	26 (43.3%)	0.40
Hemoglobin (g/dL)	13.9 ± 1.7	13.4 ± 1.7	0.027
Hematocrit (%)	41.7 ± 4.4	40.2 ± 4.7	0.03
WBC (mm3)	6596 ± 1732	7096 ± 2041	0.07
Platelet (×103/mm3)	239.8 ± 60.0	219.3 ± 46.7	0.002
ESR (mm/h)	14.6 ± 13.3	21.3 ± 17.3	0.005
Fibrinogen (mg/dL)	292.4 ± 69.6	318.6 ± 58.2	0.002
Fasting glucose (mg/dL)	106.4 ± 28.6	117.0 ± 47.5	0.011
HbA1c (%)	5.9 ± 0.9	6.2 ± 1.0	0.014
Cholesterol (mg/dL)	191.4 ± 39.0	186.3 ± 52.9	0.35
LDL cholesterol (mg/dL)	121.1 ± 31.2	121.3 ± 42.5	0.95
HDL cholesterol (mg/dL)	48.8 ± 11.2	43.2 ± 9.2	< 0.001
Triglyceride (mg/dL)	156.5 ± 88.7	171.1 ± 115.2	0.24
BUN (mg/dL)	16.6 ± 5.8	16.8 ± 5.4	0.76
Creatinine (mg/dL)	1.07 ± 0.3	1.12 ± 0.3	0.28

MACE: Major adverse cardiovascular events; MI: Myocardial infarction; CHD: Coronary heart disease; WBC: White blood cell count; ESR: Erythrocyte sedimentation rate; HbA1c: Glycated hemoglobin; LDL: Low-density lipoprotein; HDL: High-density lipoprotein; BUN: Blood nitrogen urea. Data in mean ± standard deviation.

### Prediction of MACE, hemorheological parameters and HDL cholesterol

Logistic regression analysis was performed to identify the association between hemorheological and glycemic parameters and the risk of MACE (Table 3). In univariate analysis, higher level of ESR (OR 1.026, p = 0.001), fibrinogen (OR 1.005, p = 0.006), fasting glucose (OR 1.008, p = 0.016), and HbA1c (OR 1.358, p = 0.007) increased the risk of MACE. However, in multivariate analysis, only ESR (OR 1.021, p = 0.013) and fibrinogen (OR 1.004, p = 0.04) were significantly associated with the risk of MACE. Also, lower level of HDL cholesterol increased the risk of MACE in both univariate (OR 0.945, p < 0.001) and multivariate analysis (OR 0.948, p = 0.001).

**Table 3:** Odds ratios for the association between hemorheological parameters, glycemic parameters and the risk of major adverse cardiovascular events

	**Univariate analysis**		Multivariate analysis*	
**Variable**	**Odds ratio (95% CI)**	**p value**	**Odds ratio (95% CI)**	**p value**
ESR	1.026 (1.011-1.042)	0.001	1.021 (1.004-1.038)	0.013
Fibrinogen	1.005 (1.001-1.008)	0.006	1.004 (1.000-1.008)	0.04
Fasting glucose	1.008 (1.001-1.014)	0.016	1.004 (0.997-1.012)	0.26
HbA1c	1.358 (1.087-1.697)	0.007	1.225 (0.890-1.684)	0.21
HDL cholesterol	0.945 (0.917-0.974)	< 0.001	0.948 (0.918-0.979)	0.001

* Age, gender, hypertension, diabetes, and cholesterol were included into the initial model. CI: Confidence interval; ESR: Erythrocyte sedimentation rate; HbA1c: Glycated hemoglobin; HDL: High-density lipoprotein.

### Prediction of CHD severity and hemorheological and glycemic parameters

Among the 53 patients of newly diagnosed CHD, 25 patients had one-vessel disease and 28 patients had multivessel disease. The patients with multivessel disease had significantly higher levels of fibrinogen, fasting glucose, and HbA1c compared to patients with one-vessel disease (Table 4). Among the hemorheological and glycemic parameters, higher level of fibrinogen (OR 1.013, p = 0.031 and OR 1.017, p = 0.032) and HbA1c (OR 2.519, p = 0.027 and OR 16.45, p = 0.015) increased the risk of multivessel CHD in both univariate and multivariate analysis, respectively (Table 5).

**Table 4:** Mean values of hemorheological and glycemic parameters according to the number of diseased coronary vessels in patients with coronary heart disease

**Variable**	**One-vessel disease (n = 25)**	**Multivessel disease (n = 28)**	**p value**
ESR (mm/h)	19.4 ± 11.6	22.8 ± 20.3	0.46
Fibrinogen (mg/dL)	300.9 ± 46.7	336.1 ± 60.3	0.021
Fasting glucose (mg/dL)	104.2 ± 23.7	133.3 ± 63.1	0.035
HbA1c (%)	5.9 ± 0.6	6.7 ± 1.3	0.014
HDL cholesterol (mg/dL)	44.0 ± 9.6	41.7 ± 8.9	0.37

ESR: Erythrocyte sedimentation rate; HbA1c: Glycated hemoglobin; HDL: High-density lipoprotein. Data in mean ± standard deviation.

**Table 5:** Odds ratios for the association between hemorheological and glycemic parameters and the risk of multivessel coronary artery disease

	**Univariate analysis**		Multivariate analysis*	
**Variable**	**Odds ratio (95% CI)**	**p value**	**Odds ratio (95% CI)**	**p value**
ESR	1.014 (0.978-1.051)	0.47	1.022 (0.975-1.071)	0.36
Fibrinogen	1.013 (1.001-1.024)	0.031	1.017 (1.001-1.033)	0.032
Fasting glucose	1.017 (0.999-1.036)	0.07	1.017 (0.997-1.038)	0.10
HbA1c	2.519 (1.111-5.712)	0.027	16.45 (1.704-158.75)	0.015
HDL cholesterol	0.972 (0.915-1.033)	0.36	0.940 (0.872-1.012)	0.10

* Age, gender, hypertension, diabetes, and cholesterol were included into the initial model. CI: confidence interval; ESR: Erythrocyte sedimentation rate; HbA1c: Glycated hemoglobin; HDL: High-density lipoprotein.

### Correlation of hemorheological and glycemic parameters with HDL cholesterol

Spearman univariate correlation analysis of hemorheological and glycemic parameters is shown in Table 6. ESR was significantly positively correlated with fibrinogen (r = 0.67, p < 0.001). Interestingly, ESR (r = 0.247, p < 0.001) and fibrinogen (r = 0.254, p < 0.001) were significantly positively correlated with HbA1c, but not with fasting glucose. In addition, HDL cholesterol was significantly negatively correlated with ESR (r = -0.079, p = 0.038), fibrinogen (r = -0.18, p < 0.001), fasting glucose (r = -0.158, p < 0.001), and HbA1c (r = -0.194, p < 0.001).

**Table 6:** Spearman rank correlation's (r) of each parameters

**Variable**	**ESR**	**Fibrinogen**	**Fasting glucose**	**HbA1c**
Fibrinogen	0.67			
	p < 0.001			
Fasting glucose	0.003	0.06		
	p = 0.94	p = 0.12		
HbA1c	0.247	0.254	0.514	
	p < 0.001	p < 0.001	p < 0.001	
HDL cholesterol	-0.079	-0.18	-0.158	-0.194
	p = 0.038	p < 0.001	p < 0.001	p < 0.001

ESR: Erythrocyte sedimentation rate; HbA1c: Glycated hemoglobin; HDL: High-density lipoprotein.

## Discussion

In the present study, we investigated the association between hemorheological, glycemic parameters and HDL cholesterol, and the clinical relevance of these factors for the prediction of MACE and CHD in outpatient population.

ESR, fibrinogen, and C-reactive protein are acute phase proteins whose concentrations increase in response to inflammation. Because chronic inflammation is involved in the progression of atherosclerosis and thrombosis, ESR and fibrinogen are elevated in cardiovascular disease and DM patients.^6,12^ Our data also showed that higher ESR and fibrinogen are independent risk factor of MACE. The ESR is controlled by the balance between pro-sedimentation factors, mainly fibrinogen, and those factors resisting sedimentation, especially the negative charge of the erythrocytes.^2^ When an inflammatory process is present, the increased concentration of fibrinogen in the blood causes RBC to stick to each other. Therefore, ESR and fibrinogen are hemorheological parameters which are represented as RBC aggregation and whole blood and plasma viscosity. In addition, our result showed that ESR was significantly positively correlated with fibrinogen.

DM is the most critical risk factor associated with cardiovascular disease and its chronic complications affect many organ systems. The precise mechanisms of diverse cellular and vascular dysfunction in DM are still being investigated. Among many mechanisms, hemorheological abnormalities are suggested to contribute to vascular dysfunction.^15^ However, there are few reports on the relationship between hemorheological factors and the status of glycemic control.^26^ Notably, our data showed that ESR and fibrinogen were significantly positively correlated with HbA1c, however not with fasting glucose. In addition, higher fibrinogen and HbA1c are independent risk factors of multivessel CHD in patients with MACE. Therefore, these data clearly indicated that poor glycemic control may cause hemorheological abnormalities and hence increase the risk of CHD.

Furthermore, our study showed that low HDL cholesterol significantly increased the risk of MACE, and HDL cholesterol was significantly negatively correlated with ESR, fibrinogen, fasting glucose and HbA1c. This result suggests an interaction between hemorheological parameters, glycemic parameters, and HDL cholesterol, which would contribute to the progression of atherosclerosis and thrombosis.

One limitation of our study is that we did not measure direct hemorheological parameters such as RBC deformability, plasma viscosity, and shear stress. In addition, the follow-up period was relatively short as compared with previous studies. Moreover, we had a small sample size, and were not able to compare our findings with those from other studies that used different screening methods for patients with MACE. However, ESR and fibrinogen are fast and inexpensive markers that can be easily measured in out-patient department. Therefore, our results based on an outpatient population provide the compelling evidence that ESR and fibrinogen are helpful for screening of high risk patients and guiding decisions on therapeutic interventions.

## Conclusion

Hemorheological abnormalities, poor glycemic control, and low HDL cholesterol are correlated with each other and could serve as simple, useful surrogate markers and as predictors for MACE and CHD in outpatients.
